# Epidemiology of isolated foot burns in children presenting to a Queensland paediatric burns centre— a two-year study in warmer climate

**DOI:** 10.1186/s41038-017-0070-3

**Published:** 2017-02-28

**Authors:** Florence Ngu, Bhaveshkumar Patel, Craig McBride

**Affiliations:** 1 Pegg Leditschke Children’s Burns Centre, Department of Paediatric Surgery, Lady Cilento Children’s Hospital, 501 Stanley Street, South Brisbane, QLD 4101 Australia; 2 Centre for Children’s Burns and Trauma Research, Queensland Children’s Medical Research Institute, University of Queensland, Queensland, Australia

**Keywords:** Epidemiology, Foot burns, Paediatric, Scald, Australia, Campfire

## Abstract

**Background:**

European studies of paediatric foot burns report scalds as the leading cause. Mechanisms of injury are different in warmer climates. We sought to characterize the mechanisms and outcomes of isolated foot burns in our population.

**Methods:**

Retrospective review of a prospectively collected database of all children aged 0–15 years presenting to a Queensland paediatric burns centre over a 26-month period. Non-parametric analyses such as the Mann-Whitney *U* and Pearson Chi-square were used.

**Results:**

There were 218 children with foot burns treated over a period of 2 years and 2 months of which 214 had complete records. There were significantly more boys than girls (*n* = 134, 62.6% cf. *n =* 80, 37.4%, *p* < 0.0001). The leading mechanism of injury was a contact burn accounting for 63.1% (*n =* 135) followed by scalds (23.8%, *n =* 51). Friction, flame and chemical burns were a minority but were significantly deeper (*p* = 0.03) and significantly more likely to require grafting (*p* = 0.04) and scar management (*p* < 0.0001) compared to contact and scald burns.

**Conclusions:**

In our population, contact burns are the most common mechanism of injury causing burns to the feet. The leading aetiology is campfire burns, which account for one-third of all burns to the feet. Prevention campaigns targeted at this population could significantly reduce the burden of morbidity from these burns. Friction, flame and chemical burns constitute a minority of patients but are deeper and more likely to require skin grafting and scar management.

## Background

Although foot burns by definition can only involve a small total body surface area (3.5%), they involve a specialized area of function and therefore can cause considerable morbidity [1].

Children are an at risk group for burns to the feet. The thinner skin of children, along with their inability to react appropriately to remove themselves from danger, makes them more susceptible to a deeper burn [2]. In the UK and the Netherlands, scalds have been reported as the commonest cause of an isolated foot burn [3, 4]. A warmer climate such as in Western Australia and Queensland, Australia, encourages an outdoor lifestyle, and burns caused by direct contact with hot ash or hot surfaces are common [2, 5].

The management of foot burns in children remains controversial. There are conflicting reports of reduced late sequelae seen in burns grafted before day 12 [6, 7]. Other authors advocate waiting as long as 3 weeks prior to grafting [8]. In our unit, we would consider grafting burns not predicted to heal by 2 weeks as the risk of hypertrophic scarring increases after this [9]. Hypertrophic scarring is the most important outcome in children since scarring can lead to contractures in a growing foot requiring reconstructive surgery years later [10].

There are few reports in the literature focusing on isolated paediatric foot burns [7, 8, 11], and those reporting on burns in warmer climates have included all areas of the body [2, 5]. The aim of this study is to define the epidemiology, mechanisms of injury, management and outcome of isolated foot burns in children treated at a tertiary pediatric burn centre in Queensland, Australia.

## Methods

### Source population

The Pegg Leditschke Children’s Burns Centre (PLCBC) is based at the Lady Cilento Children’s Hospital (LCCH), in Brisbane, Australia. It provides inpatient and outpatient care to children from northern New South Wales and Queensland, treating approximately 800 new burns annually.

### Database

Ethics approval was obtained prior to commencing this study from the Children’s Health Services Human Research Ethics Committee (HREC/16/QRCH/66). Data were obtained from the Queensland Paediatric Burns Registry, a prospectively collected database that was established to facilitate such research. At the time of admission, information surrounding the events of the burn is collected with the consent of the child’s parent or guardian. Depth of burn is assessed by one of five consultant paediatric burn surgeons according to the Shakespeare classification as superficial, superficial partial thickness, deep partial thickness or full thickness [12]. When the burn contains mixed depths, it is coded as the deepest element. Additional information regarding need for grafting and length of treatment is recorded in the database.

### Data collection and analysis

Supplementary information regarding outcomes and complications was obtained from medical records. Data analysis was carried out using SPSS for Mac Version 23 (IBM Corporation, Armonk, NY, USA). Appropriate non-parametric tests were used, as we did not anticipate a normally distributed data set. For the same reason, medians and interquartile ranges, rather than means and standard deviations, have been used. A *p* value below 0.05 was considered significant.

### Treatment

Blisters were deroofed early with damp cotton gauze to loosen and remove the blistered skin. All burns were dressed with either Mepitel® (Mölnlycke, Frenchs Forest, NSW, Australia) and Acticoat™ (Smith & Nephew, Hull, UK) or Mepilex® Ag (Mölnlycke, Frenchs Forest, Australia). The surgeon assessing the wound determines frequency of dressing change, typically once or twice a week depending on the nature of the burn and also family logistics. In our unit, we would consider grafting burns not predicted to heal by 2 weeks as the risk of hypertrophic scarring rises after this [9]. Patients are treated as outpatients where possible, and early ambulation is permitted, with physiotherapy input. All burns are assessed by an occupational therapist to determine whether scar management with compression garments and/or silicone is required. For the purposes of this study ‘scar management’ was any patient receiving silicone products or compression garments.

## Results

### Demographics

From January 2013 to March 2016 inclusive, 218 (8.4%) children were treated for isolated foot burns from a total of 2600 children receiving treatment for burns. There were 4 patients in whom data were incomplete, due either to parental limitations on data use or to incomplete data collection. These 4 patients have been excluded from data analysis. Of the remaining 214 patients, the median age was 30.3 months (range 0.6–178 months). There were significantly more boys than girls (61.4% cf. 36.6%, *p* < 0.0001, one-sample binomial test). There were 22 (10.3%) children who required split-thickness skin grafts. Overall, 59 (27.6%) of children required scar management. Demographic data are grouped by mechanism of injury in Table 1 and burns characteristics and outcomes in Table 2.

**Table 1:** Demographics of isolated foot burns by mechanism of injury

	Total group		Mechanism											
			Contact		Scald		Friction		Flame		Chemical		Other	
Patients, *n* (%)	214	100.0%	135	63.1%	51	23.8%	16	7.5%	5	2.3%	5	2.3%	2	0.9%
Gender, *n* (%)														
ᅟMale	134	62.6%	91	67.4%	27	52.9%	9	56.2%	5	100.0%	1	20.0%	1	50.0%
ᅟFemale	80	37.3%	44	32.5%	24	47.1%	7	43.8%	–	–	4	80.0%	1	50.0%
Median age months (IQR)	30.3	0.6–178	30.3	9.4–172.8	22.1	1.6–174.5	70.5	15–178	136.4	39.3–176.7	15.6	0.6–84	51.5	33.2–70.5
Age groups, *n* (%)														
ᅟ<1 year	80	37.3%	47	34.8%	28	54.9%	2	12.5%	–	–	3	60.0%	–	–
ᅟ1–<4 years	55	25.7%	40	29.6%	9	17.6%	3	18.8%	1	20.0%	1	20.0%	1	50.0%
ᅟ4–<11 years	60	28.0%	42	31.1%	8	15.7%	7	43.8%	1	20.0%	1	20.0%	1	50.0%
ᅟ11–15 years	19	8.9%	6	4.4%	6	11.7%	4	25.0%	3	60.0%	–	–	–	–

*IQR* interquartile range

**Table 2:** Burn characteristics and outcomes of isolated foot burns by mechanism of injury

	Total group *n* (%)		Mechanism											
			Contact *n* (%)		Scald *n* (%)		Friction *n* (%)		Flame *n* (%)		Chemical *n* (%)		Other *n* (%)	
Depth of burn														
ᅟSuperficial	7	3.2%	5	3.7%	1	2.0%	-		1	20.0%	-		-	
ᅟSPT	111	51.9%	71	52.6%	31	60.8%	3	18.8%	2	40.0%	3	60.0%	1	50.0%
ᅟDPT	82	38.3%	51	37.8%	18	35.3%	9	56.2%	2	40.0%	1	20.0%	1	50.0%
ᅟFull thickness	14	6.5%	8	5.9%	1	2.0%	4	25.0%	-		1	20.0%	-	
Trips to OT	42	19.6%	31	23.0%	3	5.9%	6	37.6%	1	20.0%	1	20.0%	-	
ᅟSkin graft	22	10.3%	14	10.4%	2	3.9%	4	25.0%	1	20.0%	1	20.0%	-	
ᅟCOD	19	8.9%	16	11.9%	1	2.0%	2	12.6%	-		-		-	
ᅟOther	1	0.4%	1 Toe amputation											
Scar management	59	27.6%	28	20.7%	13	25.5%	13	81.3%	2	40.0%	3	60.0%	1	50.0%

*SPT* superficial partial thickness

*DPT* deep partial thickness

*OT* operating theatre

*COD* change of dressing

- no patients

### Mechanism of burn

The commonest mechanism of injury was contact (*n =* 135, 63.1%), followed by scald (*n =* 51, 23.8%). The remainder were friction (*n =* 16, 7.5%), flame (*n =* 5, 2.3%) or chemical (*n =* 5, 2.3%) burns. There were two injuries classed as ‘other’ where the mechanism was not known, but the presentation was of the appearance of a burn injury and therefore treated as such. The single most common aetiology in this cohort was a campfire burn from coals or hot ash. As a single mechanism, this was responsible for 31.8% (*n =* 68) of all isolated foot burns. Coals or hot ash made up half of all contact injuries and was a more common mechanism than scalds from all causes (23.8%, *n =* 51). No other single mechanism accounted for more than 7% of this cohort.

**Fig. 1:**
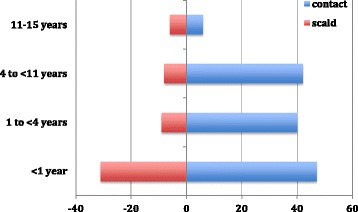
Age distribution of contact burns compared to scald burns

There was a significant difference in age distribution of the scald burn group relative to the contact burn group. Contact burns were of all ages, whereas scald burns occurred predominantly in infants aged under 12 months (*p* = 0.02, Mann–Whitney *U*), Fig. 1.

### Depth of burn

There were no significant differences in depth of burn between the scald and the contact group. Friction, flame and chemical injuries grouped together had an increased depth of burn compared with contact or scald burns (*p* = 0.03, Kruskal-Wallis).

### Dressings used for treatment

Our centre uses two different dressings—Mepitel® (Mölnlycke, Frenchs Forest, NSW, Australia) and Acticoat™ (Smith & Nephew, Hull, UK) or Mepilex® Ag (Mölnlycke, Frenchs Forest, Australia) [13]. The former dressing is changed either once or twice weekly. The latter dressing is changed twice weekly. Choice of dressing is determined by the surgeon with reference to an established protocol within the centre and by the practicalities of ensuring a robust dressing in a small foot. We were unable to determine whether one was superior to the other as there was crossover between dressings and no equivalence between groups with respect to dressing used.

### Grafting and scar management

All children who were grafted received split-thickness grafts with one exception. The exception was a child who sustained an extensive contact burn to the sole of her foot, including under all the toes. She received a full thickness graft to treat early and aggressive plantar toe contractions.

Flame, friction and chemical burns grouped together had a significantly higher risk of requiring grafting compared to the leading aetiologies of contact or scald burns (16/135, 11.9% contact; 2/51, 3.9% scald; 6/26, 23.1% other; *p* = 0.0374, Pearson Chi-square). This group also had significantly higher numbers enter scar management compared to contact or scald burns (28/135, 20.7% contact; 13/51, 25.5% scald; 18/24, 75% other; *p* < 0.0001, Pearson Chi-square).

## Discussion

The few studies investigating isolated foot burns in children have reported scalds as the most common mechanism. These studies are from the colder climates of the UK [3] and the Netherlands [4]. In our ‘sunshine state’ of Queensland, Australia, contact burns (63.1%) are far more common than scalds (23.8%). The leading aetiology was contact with hot coals/ashes, followed by contact with hot ground or a hot surface, reflecting the outdoors lifestyle. Burns due to sun-heated surfaces are unique to hot climates [2, 14].

Contact burns and scald burns constitute the vast majority of foot burns treated by our unit (186/214, 86.9%). There were no significant differences in depth of burn at presentation for these two groups. There were no differences in outcomes in terms of skin grafting or scar management between children with scald or contact burns. This was surprising, as we expected coals and hot ashes to cause more severe injuries than scalds. We speculate the absence of significant outcome differences between these groups may reflect the distribution of the burn, with a scald burn occurring from a spill onto the thinner skin of the dorsum of the foot, compared to a relatively hotter contact burn affecting the thicker skin on the sole of the foot. Thickness of skin injured may, in effect, be compensating for differences in heat of the burning agent. The finding that friction, flame and chemical burns are more likely to require grafting may reflect a deeper burn on the thinner skin on the dorsum and sides of the foot. Unfortunately, our database does not sub-classify foot burns into dorsal or plantar burns, so we were unable to see if this speculation was true. There have been reports that burns to the sole of the foot have heal with conservative management and without sequelae [15, 16]. This has not been our experience, with 28/135 (20.7%) of our contact burns group requiring scar management either subsequent to skin grafting (14/28) or following delayed healing with dressings alone.

It can be argued that in children the most important outcome of burn management is not grafting but the development of hypertrophic scarring. The natural tendency for scars to contract combined with the normal growth of a child accelerates contracture deformity [17] and can require years of reconstructive surgery until growth ceases [10]. Whilst contact burns predominate in our cohort, it is the friction, flame and chemical burns that are significantly more likely to enter scar management and therefore are most at risk of developing hypertrophic scarring and contractures. Within the time period of this study, only one child underwent surgery for release of scar contractures. There are other children in our unit, outside this cohort, who have had contracture releases for foot burns. Since contractures can develop over years [10], long-term follow-up will be required to ascertain the rates of corrective contracture surgery.

There was a significant difference in the age distribution between the group of children who sustained contact burns, who were from all age groups, and those who sustained scald burns, who were infants. This observation can help target prevention campaigns. The leading cause of contact burns is hot coals or ashes from campfires, which represent one in three of all our isolated foot burns. Our unit has previously reported the dangers and long-term sequelae from campfire burns [18]. Unfortunately, it remains the leading aetiology of isolated foot burns a decade later. Given that the age distribution of children affected is wide-ranging, targeting schools and child-care facilities particularly before holiday periods is an important public health initiative that has the potential to reduce the burden of isolated foot burns by nearly one-third in our unit. Education programmes also need to target campers about the importance of extinguishing their fires with water and not merely covering them with sand in order to lower the temperature quickly and adequately to prevent these burns [19].

## Conclusions

Isolated foot burns in the Northern Hemisphere climates are most commonly scald burns. In the hotter Queensland climate, contact burns predominate. Campfire burns from hot coals or ashes represent one-third of all isolated foot burns in our patient population. These occur in children of all ages and therefore prevention campaigns need to be targeted at all children.

The greatest risk of morbidity in our study was from burns sustained by friction, flame or chemical injury. Foot burns in children with scarring following healing require long-term follow up as the foot grows. Whilst foot burns are a small proportion of all burns, they represent significant morbidity to patients and families and a considerable workload to paediatric burns units.

## Abbreviations

LCCH: Lady Cilento Children's Hospital
PLCBC: Pegg Leditschke Children’s Burns Centre
